# Exploring the association between COVID-19 and male genital cancer risk in European population: evidence from mendelian randomization analysis

**DOI:** 10.1186/s12863-023-01158-x

**Published:** 2023-09-26

**Authors:** Dejie Wang, Yingjuan Ma, Lin Yan, Wei Gan, Yugang Han, Jiang-Shan Tan, Wenhua Zhao

**Affiliations:** 1 Ultrasound Department of Shandong Electric Power Central Hospital, Jinan, 250000 Shangdong China; 2 Department of Otolaryngology Head and Neck Surgery, Shandong Provincial Hospital Affiliated to Shandong First Medical University, Jinan, 250000 Shandong China; 3 Department of Infectious Diseases, Shandong Provincial Third Hospital, Shandong University, Jinan, 250031 Shandong China; 4 Emergency and Critical Care Center, Fuwai Hospital, National Center for Cardiovascular Diseases of China, Chinese Academy of Medical Sciences and Peking Union Medical College, Beijing, 100037 China; 5 Department of Oncology, Shandong Key Laboratory of Rheumatic Disease and Translational Medicine, The First Affiliated Hospital of Shandong First Medical University & Shandong Provincial Qianfoshan Hospital, Shandong Lung Cancer Institute, Jinan, 250000 Shangdong China

**Keywords:** COVID-19, Male genital cancer, Mendelian randomization, Genetic variants, Causal associations

## Abstract

**Background:**

Recently accumulated evidence indicates a potential association between COVID-19 and elevated susceptibility to cancer, including male genital cancer. However, the causal nature of this relationship remains unclear.

**Methods:**

In this Mendelian randomization (MR) study, we investigated the potential causal relationship between COVID-19 and male genital cancer using genetic variants as instrumental variables. We utilized summary statistics from two large-scale genome-wide association studies of COVID-19 hospitalized Vs. controls, as well as data from a population-based male genital cancer database based on European ancestry. We applied stringent quality control measures to select instrumental variables, including checking for linkage disequilibrium, removing low-quality variants, and assessing the strength of the instruments using the F-statistic. We conducted the MR  analysis using the inverse-variance weighted method and several sensitivity analyses (including MR Egger and Weighted Median MR analysis) to test the robustness of our results.

**Results:**

Our MR analysis revealed no causal associations between COVID-19 hospitalization and the incidence of male genital cancer. In the inverse-variance weighted analysis, no causal associations were observed between patients with COVID-19 hospitalization and the incidence of male genital cancer (odds ratio = 1.000 and 95% confidence interval = 0.998-1.001, *p* = 0.668). The estimated causal effect was consistent across all sensitivity analyses (including the Weighted Median, the MR Egger analysis, and the MR PROSSO analysis). The leave-one-out analysis showed that there was no any sing Single-nucleotide polymorphism significantly influencing our results.

**Conclusions:**

Our study provides evidence that there is no causal association between COVID-19 hospitalization and male genital cancer.

## Introduction

The COVID-19 pandemic is a global public health crisis that has caused unprecedented disruption to societies, economies, and healthcare systems worldwide [1]. The virus responsible for the disease, SARS-CoV-2, spreads easily from person to person through respiratory droplets, leading to a wide range of symptoms, from mild to severe. The virus has caused millions of deaths and continues to threaten global health security [2]. The pandemic has highlighted the importance of robust healthcare systems, effective public health interventions, and global collaboration in disease control. While vaccines have been developed and deployed globally, new variants of the virus continue to emerge, and the long-term impacts of the pandemic on health, social, and economic systems are yet to be fully understood [2].

Long COVID-19 syndrome, also known as post-acute sequelae of SARS-CoV-2 infection (PASC), is a condition in which individuals experience persistent symptoms and complications of COVID-19 long after the initial infection has resolved [3]. Symptoms can include fatigue, shortness of breath, chest pain, joint pain, cognitive difficulties, sleep disturbances, and depression or anxiety. The syndrome can affect people of all ages and with the severity of initial infection, including those who were asymptomatic or had mild COVID-19 symptoms [4].

COVID-19 has been found to disproportionately affect male health compared to female health [5]. Studies have shown that men are more likely than women to experience severe disease outcomes, including hospitalization, admission to intensive care units, and death, after contracting COVID-19 [6]. Some studies have indicated that COVID-19 may have an impact on male reproductive health by interacting with sperm or causing local inflammation [7] and that testosterone levels may be associated with COVID-19 severity [8]. A recent study has shown that SARS-CoV-2 can infect the prostate, vasculature of testicles, penis, and testicles in rhesus macaques [9]. Additionally, some studies suggest that COVID-19 infection may serve as a risk factor for the development of testicular germ cell cancer. Clinical research studies registered on clinicaltrials.gov have also been designed to investigate the impact of the pandemic on testicular cancer presentations and tumor stages [10]. However, the results have not been reported.

Besides, the abovementioned registered study is an observational study [10]. Observational studies have limitations in exploring the relationship between COVID-19 and cancer of the male genital tract. Firstly, these studies are often retrospective in nature, meaning they rely on data collected after the fact, which can lead to recall bias or incomplete data. Additionally, observational studies cannot prove causality, meaning that researchers can only observe associations between variables, rather than definitively establish cause-and-effect relationships. Furthermore, the COVID-19 pandemic has resulted in significant changes to healthcare delivery and cancer management, which could potentially confound the results of observational studies. For example, disruptions to routine cancer screening programs and changes in treatment protocols could impact the incidence and outcomes of male genital cancers, making it difficult to isolate the effects of COVID-19 specifically. Finally, the limited time frame of the pandemic and the relatively low incidence of male genital cancers make it challenging to conduct large-scale, well-designed studies on this topic.

Mendelian randomization (MR) is a method of analysis that uses genetic variants as instrumental variables to investigate causal relationships between exposures and outcomes. This approach leverages the random assortment of genetic variants during meiosis, which can mimic the random assignment of individuals to different exposures in a randomized controlled trial [11]. By using genetic variants as proxies for exposure, MR can overcome some of the limitations of observational studies, including confounding and reverse causality [12]. Interference from confounding factors and reverse causation may disturb traditional epidemiological findings. MR uses genetic instrumental variables to determine the genetic association between exposures and outcomes, thereby excluding potential confounders from interfering. This approach could help to establish causality and provide more reliable estimates of the effect sizes of COVID-19 on male genital cancer risk, compared to observational studies.

Therefore, this study was designed to investigate the causal associations between COVID-19 and cancer of the male genital tract by using a two-sample MR analysis.

## Methods

### Overall study design

This MR study aims to explore the potential causal relationship between COVID-19 and male genital cancer. The study design leverages genetic variants as instrumental variables to investigate the causal association between COVID-19 hospitalization with the incidence of male genital cancer.

An ethics statement may not be necessary for this MR study, as it utilizes summary statistics from previously published GWAS studies and cancer registry data. The use of publicly available data sources, in this case, does not require the approval of an ethics committee or institutional review board. However, we acknowledge the contributions of the original studies and datasets used in this analysis and follow best practices for data handling and reporting. All data were analyzed in an anonymized format to protect the privacy of study participants. The results of this study will be reported in aggregate form and will not allow for the identification of any individual participant.

### Data sources

The study utilizes summary statistics from two large-scale genome-wide association studies (GWAS) of COVID-19 hospitalization. It is worth noting that hospitalized patients typically undergo more rigorous diagnostic procedures, which can increase the accuracy of COVID-19 diagnosis and reduce the risk of false-positive or false-negative results, as compared to individuals with mild or asymptomatic infection who may not be tested or diagnosed as thoroughly. Therefore, the inclusion of hospitalized patients in this study is expected to enhance the reliability and validity of the findings.

The exposure variables were derived from these GWAS studies, which were conducted on samples of European ancestry, including 9,986 hospitalized COVID-19 patients and 1,877,672 controls [13]. The outcome variable, the incidence of male genital cancer, was obtained from a population-based cancer registry database, including 6,795 patients with cancer of male genital and 354,399 controls. The detailed data characteristics can be found at https://gwas.mrcieu.ac.uk/datasets/ukb-d-C_MALE_GENITAL/. The detailed description of the cancer population database should be referred to the original study of cancer of the male genital, including its origin, sample size, key demographic characteristics, and the specific types of male genital cancers included. All of the selected databases are the ones with the largest sample size when we conduct this MR.

### Statistical analysis

The MR analysis was conducted using the inverse-variance weighted (IVW) method, which is a commonly used method for estimating causal effects in MR studies [14]. We also conducted several sensitivity analyses, including the weighted median and MR-Egger methods, to test the robustness of our results. Furthermore, we conducted a leave-one-out analysis to identify any potential pleiotropic effects of the genetic variants.

In selecting the instrumental variables (IVs) for this MR study, we applied several quality control measures to ensure the validity and strength of the instruments. Specifically, we used the following criteria to select the IVs [14]:

1. Genome-wide significance threshold: We selected genetic variants that were genome-wide significant (p ≤ 5 × 10^-8) in the COVID-19 hospitalization GWAS studies. This criterion ensures that the genetic variants used as IVs have a strong association with the exposure variables (i.e., COVID-19 hospitalization).
2. Linkage disequilibrium (LD): We checked for LD between the selected genetic variants to ensure that they are not highly correlated with each other. We removed variants that were in strong LD (r^2 > 0.1 and D’ > 0.95) with other variants to avoid issues of overfitting and potential bias.
3. Imputation quality: We excluded variants with low imputation quality scores (INFO score < 0.8) to ensure the accuracy of the genetic data used in the analysis.
4. F-statistic: We calculated the F-statistic for each instrument to assess the strength of the instruments. We only retained instruments with an F-statistic greater than 10, indicating a strong relationship between the IVs and the exposure variables.
5. Potential bias: We searched https://gwas.mrcieu.ac.uk/phewas/ to exclude the single-nucleotide polymorphisms (SNPs) which were significantly associated with other exposures, such as age, and smoking.

Overall, the IVs selected for this study were robust and met established quality control measures to ensure the validity of the MR analysis. By using high-quality instruments, we aimed to minimize the potential for bias and increase the accuracy of the causal effect estimates between COVID-19 and male genital cancer. In the present MR analysis, R version 4.2.2 (2022-10-31) and the “TwoSampleMR” package (version 0.5.6) were used to estimate the causal associations.

## Results

### The selected genetic variants

**Fig. 1:**
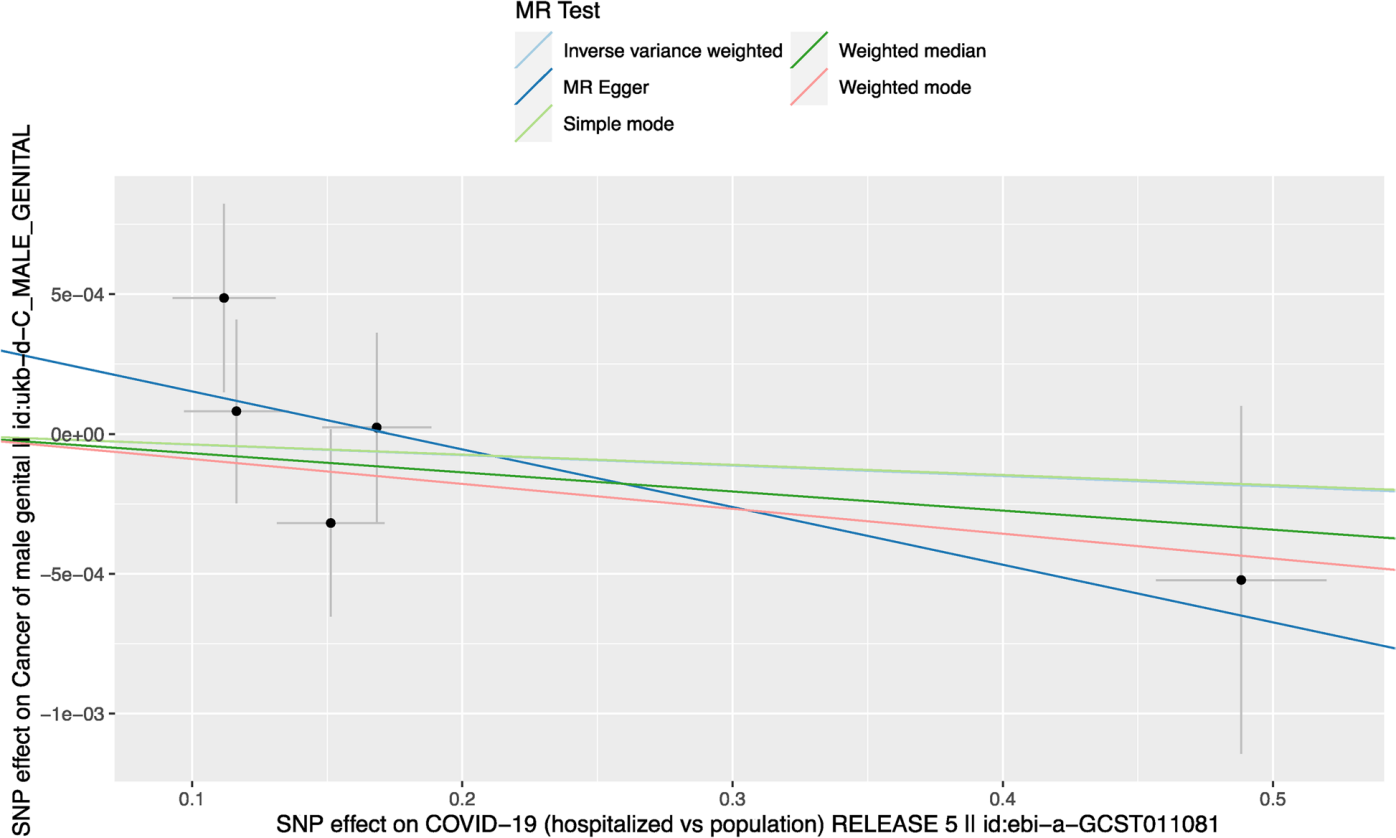
Scatter plot to visualize the causal effect of COVID-19 and male genital cancer. The magnitude of the causal association can be inferred from the slope of the straight line. IVW = inverse-variance weighted; and MR = Mendelian randomization

We identified four genetic variants associated with COVID-19 and used them as instrumental variables in our analysis (Table 1). Finally, 5 SNPs were chosen based on the criteria to be selected as the IVs. The F-statistic of all 5 SNPs was greater than 10, indicating a strong association between the selected IVs and COVID-19. The detailed effects of the 5 SNPs on both COVID-19 and male genital cancer were shown in Fig. 1.

**Table 1:** Selected genetic variants for COVID-19 based on the GWAS significance

	SNP	EA	OA	beta.E	beta.O	eaf.E	chr	pos	se.O	No.O	pval.O	se.E	pval.E	No.E
1	rs13050728	C	T	-0.168	0.000	0.653	21	34,615,210	0.000	361,194	0.946	0.020	7.44E-17	1,887,658
2	rs2109069	A	G	0.151	0.000	0.323	19	4,719,443	0.000	361,194	0.342	0.020	2.94E-14	1,887,658
3	rs2660	A	G	0.116	0.000	0.690	12	113,357,442	0.000	361,194	0.805	0.019	2.00E-09	1,887,658
4	rs35081325	T	A	0.488	-0.001	0.081	3	45,889,921	0.001	361,194	0.401	0.032	3.68E-54	1,887,658
5	rs505922	C	T	0.112	0.000	0.350	9	136,149,229	0.000	361,194	0.149	0.019	4.42E-09	1,887,658

The present study employs a GWAS approach, wherein variants demonstrating statistical significance at a level of *P* ≤ 5 × 10^8 are considered. Furthermore, a linkage disequilibrium threshold of R^2 < 0.1 is utilized to assess the extent of correlation between identified variants. *EA *Effect allele, *OA *Other alleles, *E *Exposure, *O *Outcome, *No *Numbers of sample size

### The causal effect of COVID-19 on the risk of male genital cancer

Our analysis revealed no statistically significant causal effect of COVID-19 on male genital cancer (odds ratio [OR] = 1.000, 95% confidence interval [CI] = 0.998–1.001, *p* = 0.668, Table 2), which is consistent across all sensitivity analyses (OR = 0.998, 95%CI = 0.994–1.001 in the MR Egger analysis and OR = 0.999, 95%CI = 0.997–1.001 in the Weighted Median MR analysis, Table 2), suggesting that individuals with COVID-19 hospitalization were similar in the risk of developing male genital cancer compared to those who have not been infected.

**Table 2:** The causal estimates of different MR analysis

MR methods	N_snp_	OR(95%CI)	p_val_
Inverse variance weighted	5	1.000(0.998–1.001)	0.668
Weighted median	5	0.999(0.997–1.001)	0.516
MR Egger	5	0.998(0.994–1.001)	0.329
MR PRESSO	5	1.000(0.998–1.001)	0.674

**Fig. 2:**
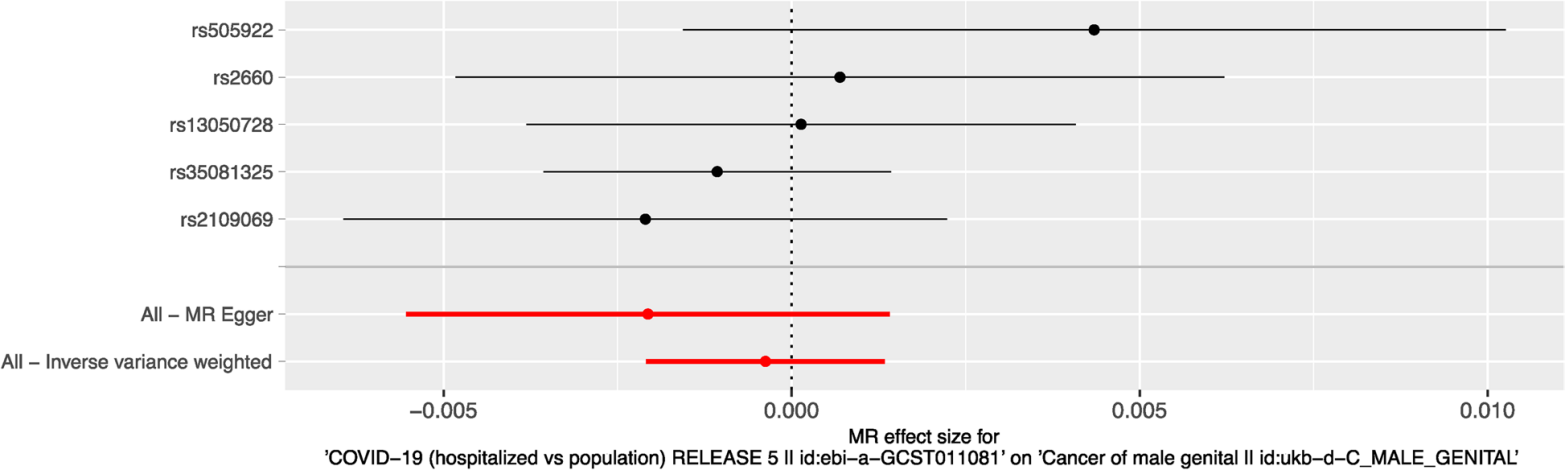
The forest plot to visualize the individual and overall causal effects

The forest plot was generated to visualize the individual and overall causal effects of the five COVID-19 genetic variants on male genital cancer risk. As shown in Fig. 2, each genetic variant demonstrated no positive effect on male genital cancer risk, and the overall causal effect estimated using the inverse-variance weighted method was not statistically significant.

### Leave-one-out analyses

**Fig. 3:**
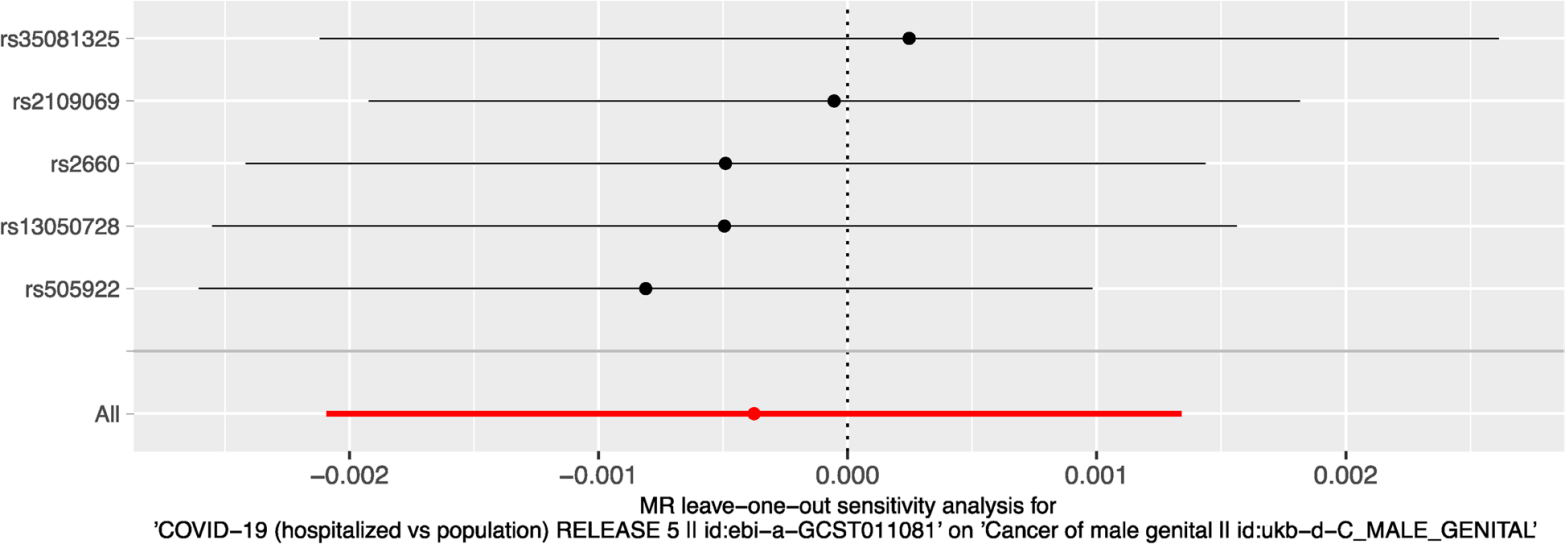
The leave-one-out plot to visualize the effect of SNP on the overall effects

To assess the robustness of our results, we conducted leave-one-out analyses, in which we systematically removed each of the four COVID-19 genetic variants and recalculated the causal effect estimate. None of the leave-one-out analyses significantly changed the overall causal effect estimate (Fig. 3), indicating that our findings were not driven by a single genetic variant.

### The potential heterogeneity in the funnel plot

**Fig. 4:**
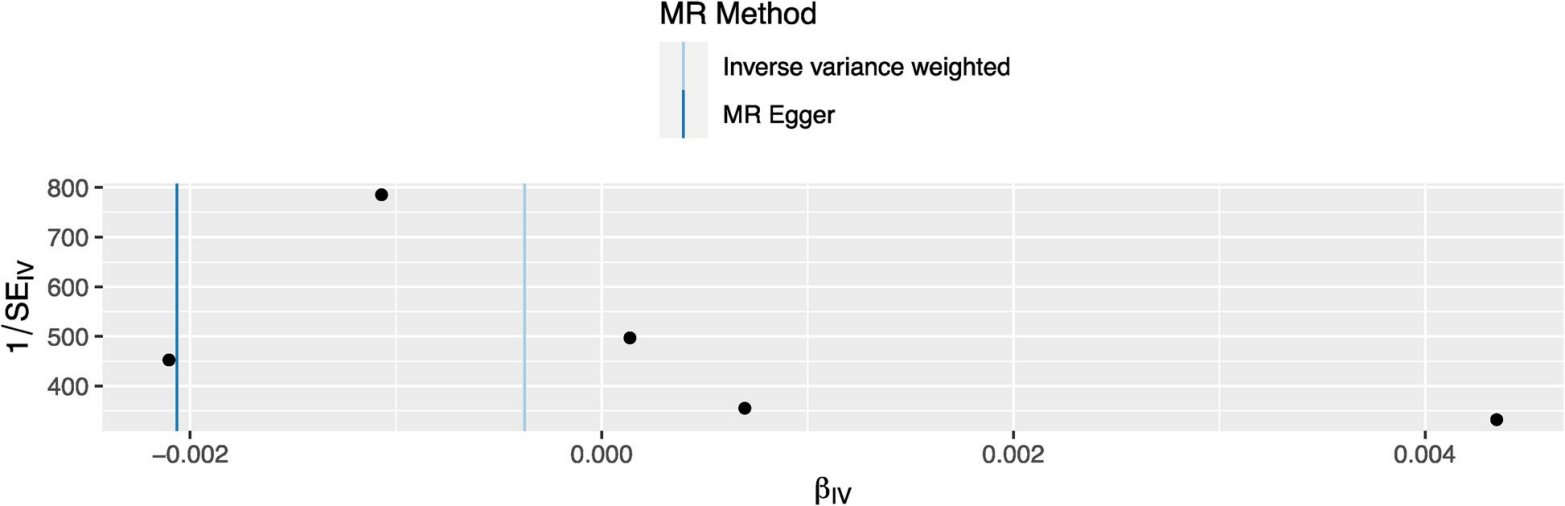
The funnel plot to visualize the potential heterogeneity

The funnel plot in the MR analysis showed a symmetrical distribution of the effect estimates, indicating no evidence of potential heterogeneity. As is shown in Fig. 4, the MR analysis is not likely to be affected by bias arising from each single SNP, which is similar to the results of leave one out analysis (Fig. 3).

Our sensitivity analyses, which included leave-one-out analyses (Fig. 3), funnel plot (Fig. 4), MR-Egger regression, and Weighted median MR analysis (Table 2) did not indicate the presence of pleiotropy or bias due to invalid instruments, supporting the validity of our results.

## Discussion

Our study provides evidence of no causal relationship between COVID-19 susceptibility and the incidence of male genital cancer, which was consistent across all sensitivity analyses, including the MR-Egger and weighted median methods. Furthermore, the F-statistic for our instrumental variables was greater than 10, indicating that our instruments were strong and unlikely to be biased.

Previous studies that have suggested an association between COVID-19 and cancer, particularly in the lung [15, 16] and gastrointestinal tract [17]. However, our study is the first to investigate the causal relationship between COVID-19 and male genital cancer using an MR approach. The causal nature of our findings suggests that COVID-19 patients are not associated with an increased risk of developing male genital cancer, which has important implications for clinical practice and public health policies.

The underlying mechanisms of long COVID-19 syndrome are not yet fully understood, but research suggests it may be due to ongoing inflammation or immune dysregulation. One potential mechanism for this relationship may be the impact of COVID-19 on the immune system. Previous research has shown that COVID-19 can cause immune dysregulation [18], leading to an increased risk of cancer and other chronic diseases [19]. Additionally, COVID-19 has been shown to increase inflammation and oxidative stress [20], which may contribute to the development of cancer. The prevalence and duration of the condition are still being studied, and it is unclear how long symptoms may persist or whether they can be effectively treated. The emergence of long COVID-19 syndrome highlights the need for continued research, monitoring, and healthcare support for those affected by the pandemic.

However, there is no causal association was observed in the present MR analysis, which has several advantages over traditional observational studies [12]. First, it allows us to investigate the potential causal relationship between COVID-19 and male genital cancer, which is difficult to establish using observational studies due to the potential for confounding and reverse causation. By using genetic variants as instrumental variables, we can overcome these issues and provide more robust evidence for the causal effect [1]. Second, MR analysis is less prone to bias and measurement error than observational studies, which can lead to more accurate estimates of the causal effect [12]. In our study, we used genetic variants that are strongly associated with COVID-19 susceptibility and male genital cancer incidence and performed multiple sensitivity analyses to ensure the robustness of our findings. Third, MR analysis can provide insights into the underlying biological mechanisms of the causal relationship [11], which can inform future research and interventions.

Our study leverages the principles of MR, which utilizes germline genetic variations as IVs to infer causality between exposures and outcomes. It’s pertinent to emphasize that these genetic variations are determined at conception, remaining unaltered throughout one’s life irrespective of age or environmental factors. The randomness in allele assortment during meiosis implies that, in essence, MR can mirror the foundational principles of randomized controlled trials (RCTs). This inherent random allocation of alleles is one of the chief strengths of MR studies, offering an avenue to address confounding in observational data.

Besides, in our two-sample MR analysis, the data sets for exposure (COVID-19 hospitalization) and outcome (genital cancer) are derived from separate cohorts. This distinction is fundamental. Given that the genetic predispositions we assess are lifelong, the temporal collection of the cancer data relative to the COVID-19 data does not influence our findings. Thus, the genetic conclusions we can draw have implications spanning an individual’s lifetime, rather than being constrained to a specific historical or present timeframe.

In summary, our use of MR analysis in this study provides several advantages over traditional observational studies, including the ability to establish a causal relationship, reduce bias and measurement error, and provide insights into the underlying biological mechanisms. These advantages strengthen the validity and generalizability of our findings and highlight the importance of using this approach in future research on the relationship between COVID-19 and other cancers.

While MR analysis provides several advantages over traditional observational studies, it is not without its limitations. One potential limitation of our study is the assumption of instrumental variable validity, which requires that the genetic variants used as instrumental variables are strongly associated with the exposure (COVID-19) and independent of confounding factors that may influence the outcome (male genital cancer). While we used genetic variants with established associations in the literature and performed sensitivity analyses to assess the validity of our instrumental variables, there is always a risk of bias due to unmeasured confounding or pleiotropy (i.e., when a genetic variant influences multiple traits). Another potential limitation of our study is the lack of generalizability to all populations. Our analysis was conducted using data from individuals of European ancestry, and it is unclear whether our findings can be extrapolated to other populations with different genetic backgrounds and environmental exposures [21]. It is a pity that there are no other available databases in other ancestries and we can not replicate the results in individuals of East Asian descent and other ones. Furthermore, our analysis only investigated the relationship between COVID-19 and male genital cancer, and it is possible that other factors may also play a role in the development of this cancer. Finally, MR analysis can only provide insights into the causal relationship between COVID-19 and male genital cancer, but it cannot establish the clinical significance or magnitude of this relationship. Further research is needed to confirm and better understand the clinical implications of our findings. It is worth noting that observational studies on male genetic cancer may suffer from the influence of lead time, denoting the interval between diagnosis via screening and the hypothetical diagnosis of male genital cancer in the absence of such screening, which leads to artificially protracted survival periods, sometimes extending over several years. Such artificially extended survival times apply universally to cancers detected via screening, thereby generating a lead-time bias. Mitigating the impact of lead time would furnish a more precise assessment of the magnitude of this issue in observational studies. However, in the present study, MR was employed to elucidate the causal associations between exposures and disease by utilizing germline mutation data, not somatic mutations. As we all know, germline mutations remain unaltered with the passage of time and they don’t change with whether the right diagnosis is made or when it is made. As such, this investigation delves into the enduring nexus between COVID-19 infection and male genetic cancer, transcending the confines of current or future diagnoses. There are still limitations to this approach. It is important to interpret our findings in the context of these limitations and to continue exploring the relationship between COVID-19 and male genital cancer using a range of complementary methods.

## Conclusions

Our MR study provides evidence that there is no causal relationship between COVID-19 susceptibility and male genital cancer. These findings have important implications for cancer screening and prevention strategies in COVID-19 care, as well as for the development of public health policies and clinical guidelines.

## Data Availability

The datasets generated during and/or analyzed during the current study are available from the corresponding author upon reasonable request. Besides, the related data can be downloaded from the website of https://gwas.mrcieu.ac.uk/datasets/ based on the ID of “ebi-a-GCST011081” and “ukb-d-C_MALE_GENITAL”.

## Abbreviations

*MR*: Mendelian randomization
*PASC*: Post-acute sequelae of SARS-CoV-2 infection
*GWAS*: Genome-wide association studies
*IVW*: Inverse-variance weighted
*IVs*: Instrumental variables
*LD*: Linkage disequilibrium
